# Can anterior repositioning splint effectively treat temporomandibular joint disc displacement?

**DOI:** 10.1038/s41598-018-36988-8

**Published:** 2019-01-24

**Authors:** Zhigui Ma, Qianyang Xie, Chi Yang, Shanyong Zhang, Yuqing Shen, Ahmed Abdelrehem

**Affiliations:** 1 Department of Oral Surgery, Shanghai Ninth People’s Hospital affiliated to Shanghai Jiaotong University, school of medicine, Shanghai Key Laboratory of Stomatology, Shanghai, P. R. China; 2 Department of Craniomaxillofacial and Plastic Surgery, Faculty of Dentistry, Alexandria University, Alexandria, Egypt

## Abstract

The aim of this study was to determine whether anterior repositioning splint (ARS) can effectively treat temporomandibular joint (TMJ) anterior disc displacement with reduction (DDwR) in juvenile Class II patients. This study investigated disc repositioning clinically and through use of MRI with 12-month follow up. Patients with skeletal Class II malocclusions and DDwR diagnosed by magnetic resonance imaging (MRI) were treated with ARS. The efficacy of ARS was assessed clinically and by means of MRI before treatment (T0), immediately after bite registration (T1), at the end of treatment (T2), and at 12 months after functional appliance treatment (T3). Improvement in TMJ pain, TMJ noises, and range of mandibular movement were assessed. MRI evaluation was based on disc-condylar relationship in parasagittal images. Seventy-two juvenile patients with 91 joints were included in this study. The average age was 15.7 years old (range, 10–20 years) at first visit. There were statistically significant reductions in TMJ pain, disability in daily life and TMJ clicking (*P* < 0.01). MRI at T2 indicated that the success rate was 92.31% (84/91), but decreased to 72.53% (66/91) at T3. The unsuccessful splint disc capture was mainly observed in late adolescence, especially over 18 years old. Using MRI results as the gold standard, we found that clinical assessment had an accuracy rate of 75.82% at 12-month follow-up. In conclusion, although success rate for ARS treatment decreased over time, both clinical findings and MRI examination indicate that the ARS is relatively effective in repositioning the DDwR, especially for patients in early puberty. However, further and larger studies are needed to evaluate the outcome with ARS.

## Introduction

Disc displacement with reduction (DDwR) of the temporomandibular joint (TMJ) is the most frequent form of temporomandibular internal derangement and involves abnormal disc-condyle relationships. The disc is displaced anteriorly relative to the condyle when the mouth is closed and can be reduced with mouth opening^1^. Anterior displacement of the disc results in TMJ clicking, joint pain and, ultimately, in condylar resorption and jaw deformity^2–4^. In a previous study, we found that anterior disc displacement in growing patients was significantly associated with decrease in condylar height and mandibular asymmetry^5^. Re-establishing a normal articular disc–condyle relationship can contribute to condylar adaptive remodelling^6^. Mehra and Wolford have reported a statistically significant reduction in TMJ pain, TMJ noises, and disability, and improvement in jaw function after disc repositioning^7^. Hence we believe that normalization of altered disc–condyle relationship should be considered in symptomatic patients to prevent serious damage to the TMJ. Because disc displacement does not correct itself spontaneously and early recapture of the reducing disc should be considered before it is severely deformed.

Functional appliances have been widely used in the field of orthodontics and dentofacial orthopaedics for the correction of mandibular retrognathia in order to stimulate mandibular growth by forward positioning the mandible during the growth period^8,9^. Another effect of functional appliance is that it can reposition condyles anteriorly to catch or ‘re-capture’ displaced discs, establishing normal disc – condyle relationships in the mandibular fossae and accelerate condylar growth^10^. However, Class I and Class III malocclusion is not suitable for bite jumping treatment because of mandibular positon. Only for skeletal Class II malocclusion with DDwR, when the mandible is repositioned forward and downward, physiological relationships between the disc and the condyles can be simultaneously achieved with the insertion of a functional appliance. Thus, we believe that functional appliance, under proper use, helps correct skeletal Class II malocclusion, and, simultaneously, facilitates capture of an anteriorly displaced disc^11–13^. However, there have been very few reports in the literature about the effect of functional treatment for DDwR companied with mandibular retrognathia.

The anterior repositioning splint (ARS) is a removable, convenient, and simple device that is commonly used for the management of DDwR. Some studies have evaluated the effect of ARS therapy on TMJ disc positon^14–17^. Preparation and placement of the ARS is usually based on clinical experience^17^. We think it is necessary to confirm ARS recapture by means of imaging immediately before splint therapy. However, few studies have used imaging modalities to ascertain disc recapture at the onset of splint treatment^13,16^.

This study aims to provide new understanding of ARS as a functional appliance for treating DDwR and coexisting mamdibular retrognathia simultaneously. We hypothesized that ARS could obtain a stable repositioning of the disc in skeletal Class II subjects with a pretreatment DDwR. The present investigation aimed at evaluating the effect of ARS treatment on disc position in patients with DDwR both clinically and with MRI.

## Patients and Methods

### Patients

The study protocol was approved by the Institutional Review Board of Shanghai Ninth People’s Hospital affiliated to Shanghai Jiao Tong University, School of Medicine (No. S9HIE 2017-348-T257). All participants signed an informed consent agreement for this study. Between November 2010 and January 2016, consecutive patients were recruited for the study from the TMJ division of Shanghai Ninth People’s Hospital affiliated to Shanghai Jiao Tong University. The inclusion criteria included: (a) the patient aged between 10 to 20 years with no gender limitation; (b) clinical diagnosis of DDwR based on the presence of reciprocal clicking^18^; (c) further confirmation of DDwR with MRI; (d) with complete dentition; (e) Class II malocclusion with at least an end-to-end molar and canine relationship. The exclusion criteria included: (a) patient had a history of functional appliance therapy, orthodontic and/or orthognathic treatment; (b) contraindications to the MRI, such as patients with a heart pacemaker or severe claustrophobia; (c) periodontal disease; (d) Class I and Class III malocclusion; (e) major psychological disorders; (f) poor compliance.

### Functional appliance

**Figure 1:**
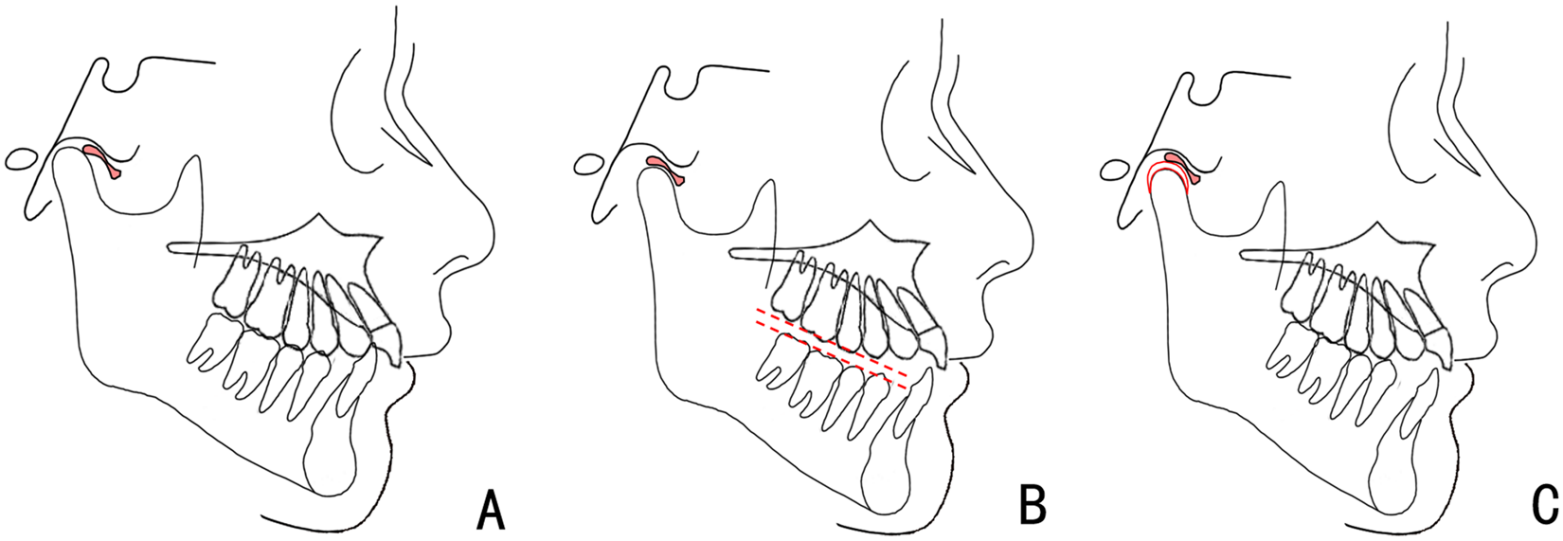
Schematic diagram shows the change of disc-condylar relationship, occlusion and facial type with maxillary ARS. (**A**) DDwR, Class II malocclusion with mandibular retrusion before treatment; (**B**) disc recapture, Class I malocclusion and improved facial profile with ARS insertion; (**C**) successful disc recapture with condylar remodelling and good occlusion after treatment.

**Figure 2:**
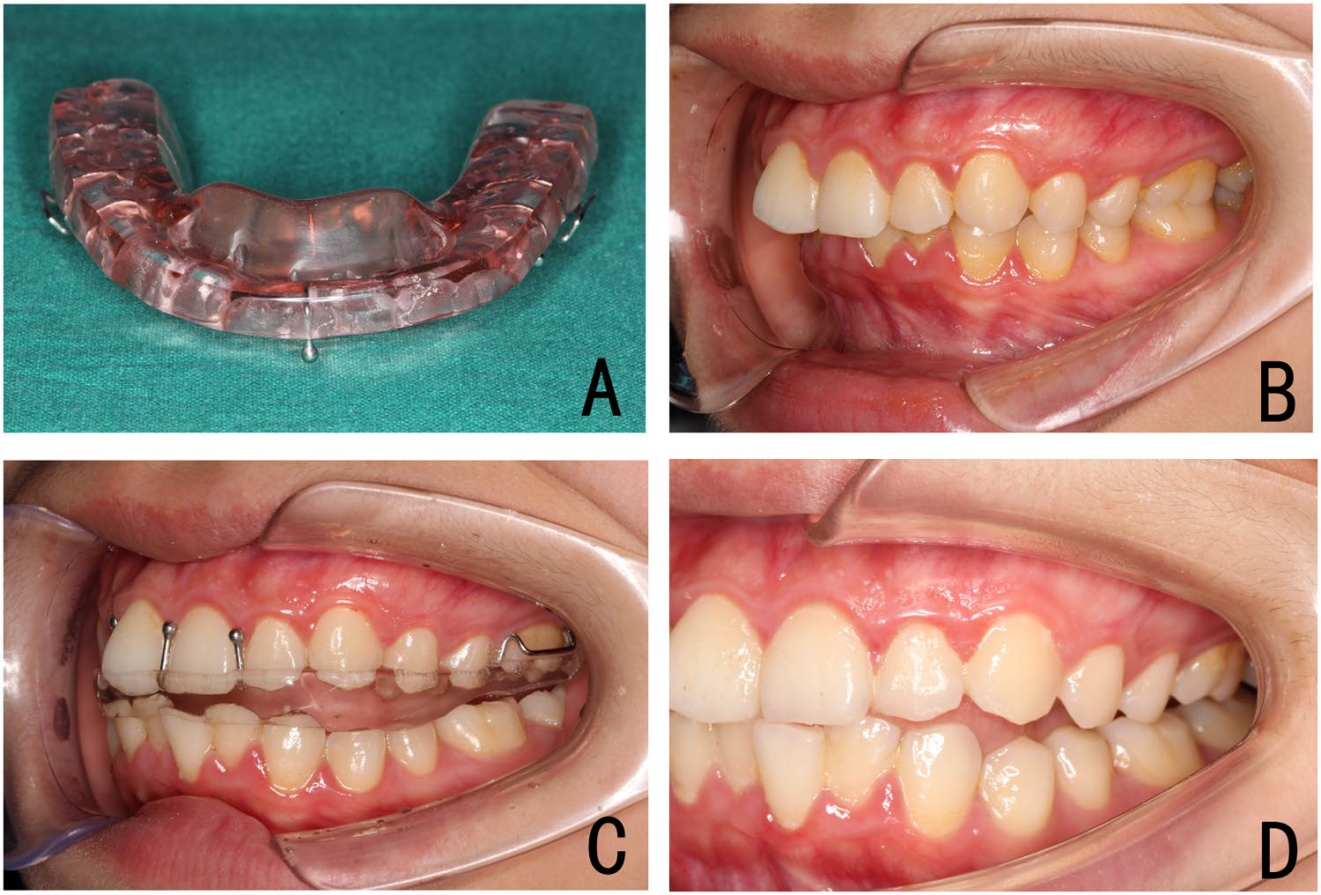
Full-coverage ARS in the therapeutic protrusive jaw position. (**A**) the bite block keeps the mandible in the anterior position; (**B–D**) occlusal re-establishment with ARS.

The initial wax construction bite was taken by advancing the mandible to an incisal edge-to-edge position and achieve a Class I or super Class I molar relationship with a 5 mm vertical opening in the premolars region (Fig. 1), where reciprocal clicking should be eliminated upon month opening. To confirm that discs were captured, the patients were scheduled for TMJ MRI with anterior repositioning occlusal registration in place before fabricating the splint. ARS with a bite block was used to stabilise the protrusive position (Fig. 2A). Patients were instructed to wear the appliance 24 hours a day except for brushing their teeth. At follow-up visits, acrylic was ground by 1 mm every 4–6 weeks from the posterior areas to clear the occlusal aspect of the lower molars and premolars, thereby encouraging vertical eruption of these teeth, settling occlusion and Class I molar relation, and for occlusal plane levelling^19,20^. Then the ARS will stay in place for another 1–3 months to maintain the mandible in a stable position. When a stable occlusal condition was re-established, and the mandible did not obviously relapse to a retrusive position after 6 weeks without the ARS, the functional treatment was considered completed (Fig. 2B–D).

### MRI of TMJ

MRI was performed using a 1.5-T scanner (SIGNA; GE Medical Systems, Milwaukee, WI, USA) with a 6 cm × 8 cm TMJ surface coil receiver on each side, according to the routine sequence^21^. MRI of the TMJs was performed at four time points: before functional treatment (T0), immediately after the insertion of bite wax (T1), at the end of functional treatment (T2), and at 12 months after completion of treatment (T3).

### Evaluations

#### Clinical evaluation

The subjects were clinically assessed for signs and symptoms according to Mehra and Wolford (7) and Kurita *et al*.^15^. The patients were asked about presence of joint clicking and pain before ARS treatment. Visual analogue scales (VAS) were used for subjective evaluation of joint pain (0 = no pain, 10 = severe pain). Disability in daily life, including jaw locking, sleep disturbance, disability on chewing and absence from work due to joint symptoms, was also scored using the same method. Objective evaluation included assessment of TMJ clicking, maximum interincisal opening (MIO), protrusive excursion (PE), left lateral excursion (LLE) and right lateral excursion (RLE). At the end of treatment, if the patient had nearly no pain or disability in daily life and there was no joint clicking or only occasional clicking during mouth opening (one or two times per day), splint capture was considered clinically successful. If the patient continued to experience pain or joint clicking, ARS treatment was judged to have failed.

### MRI assessment

**Figure 3:**
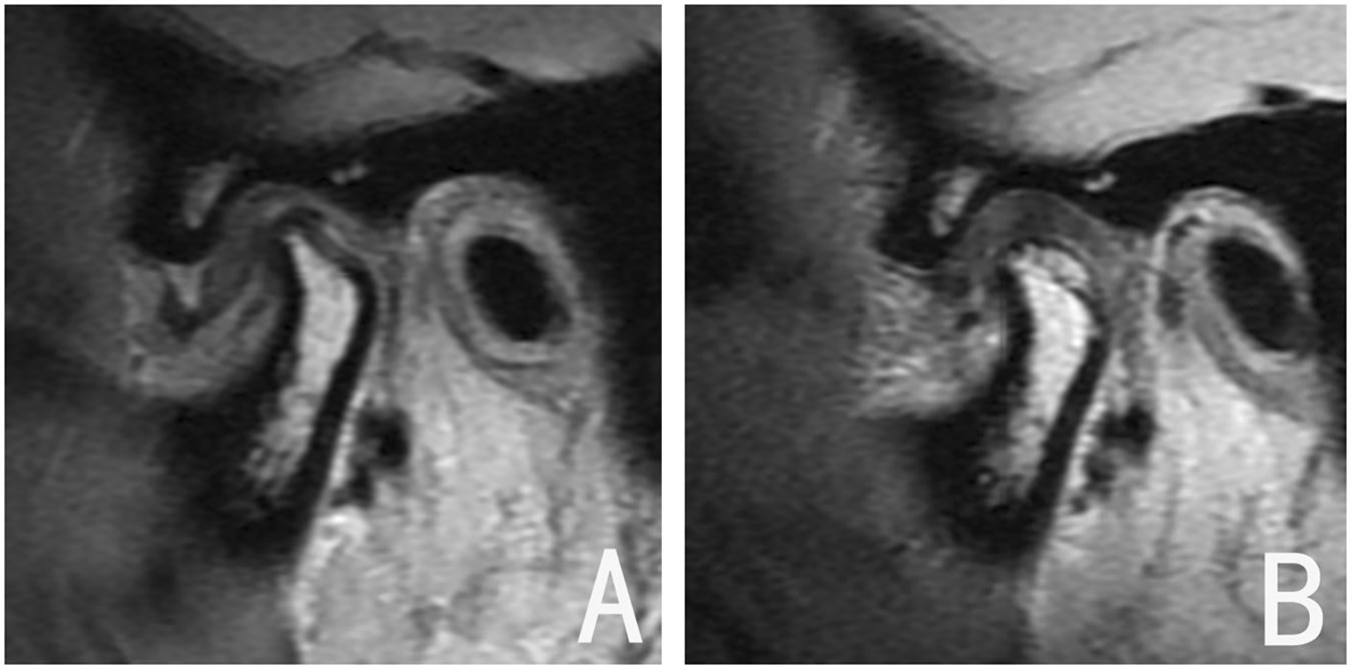
Joint with excellent outcome. (**A**) DDwR before treatment; (**B**) disc recapture after functional appliance treatment, and the new bone apposition on the posterosuperior region of the condyle.

**Figure 4:**
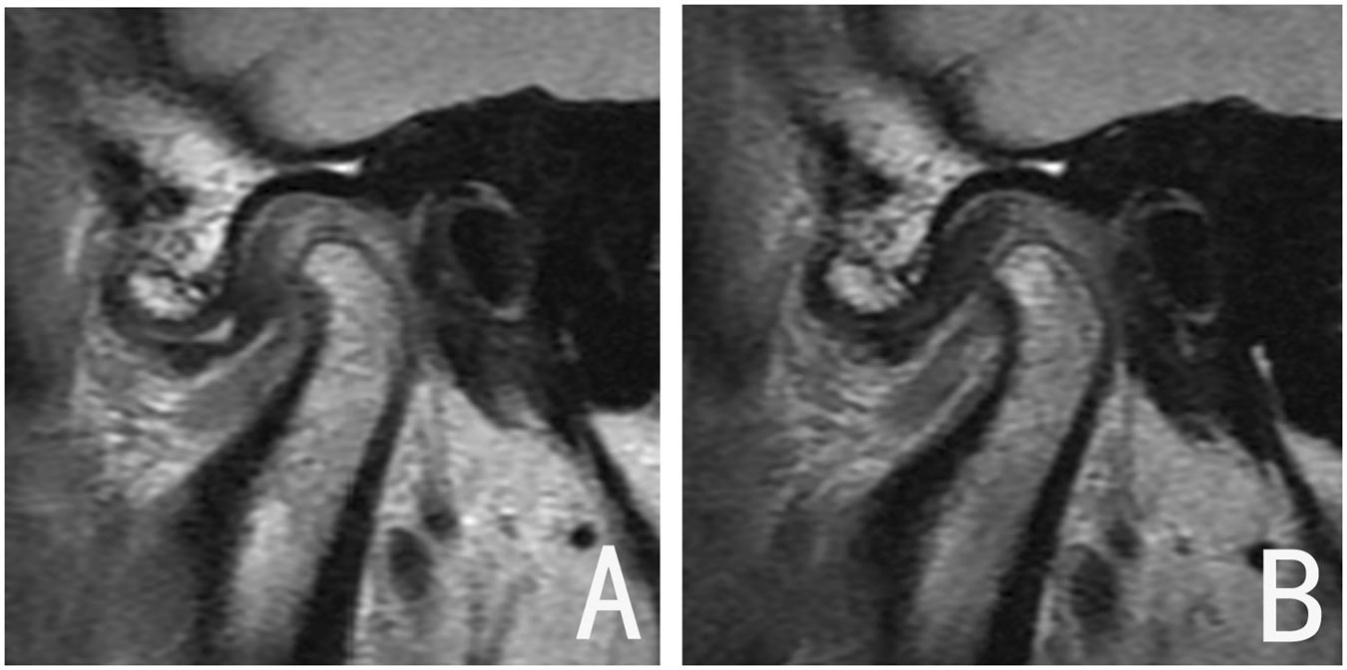
Joint with good outcome. (**A**) DDwR before treatment; (**B**) disc recapture after treatment, with no remarkable condylar remodeling.

**Figure 5:**
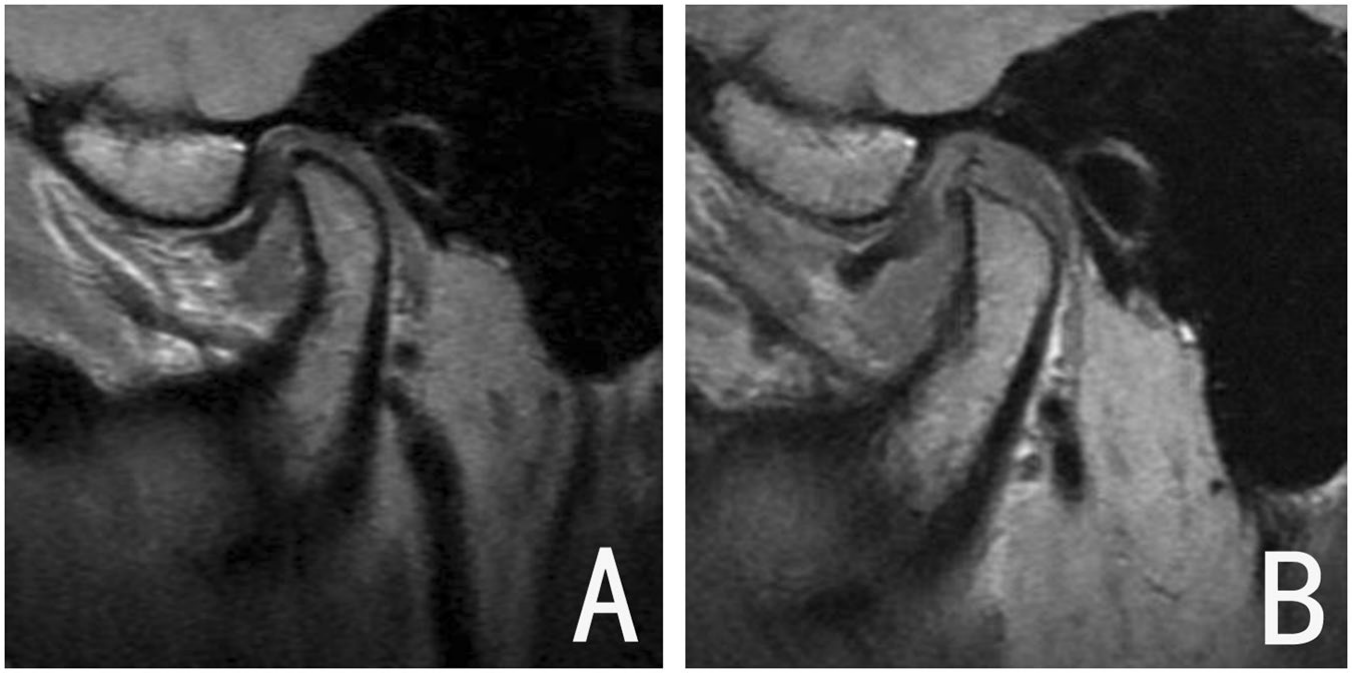
Joint with poor outcome. (**A**) DDwR before treatment; (**B**) the disc was not captured after treatment.

Evaluation of MR images was based on the location of the disc relative to the condyle in the parasagittal image. A normal disc-condyle relationship with reparative condylar change (new bone formation on the condyle) was considered an excellent outcome (Fig. 3); mild disc displacement accompanied by a disc-like bilaminar zone, or a normalized disc-condyle relationship without reparative condylar change, was considered a good outcome (Fig. 4); and persistent anterior disc displacement was considered treatment failure (Fig. 5). Excellent and good evaluations were regarded as radiographic successes.

### Statistical analysis

The data were processed using the SPSS 17.0 (SPSS, Inc, Chicago, IL). Repeated measures analysis of variance with post hoc Bonferroni test was used to test differences before treatment, after the treatment, and at follow-up. With regard to nominal data, McNemar χ^2^ test was used to compare pretreatment and posttreatment differences. Method error was calculated by means of a variance analysis. Statistical significance was determined at the 1% and 5% levels of confidence..

## Results

The mean age of onset of DDwR was 15.7 years (range from 10 to 20 years), and the mean duration of symptoms was 8.3 months. On average, patients received 5.7 months (ranged, 1 to 24 months) of nonsurgical therapy, including treatment with medications, before being treated with ARS. There were 78 patients (58 females and 20 males) prepared to receive ARS for treating class II malocclusion accompanied with DDwR, 3 of them who complained of discomfort with the appliance and stopped treatment early (1 female and 2 male), and 3 of those in whom MRI showed anteriorly displaced disc after insertion of bite registration, were excluded (2 females and 1 male). As a result, the final study sample comprised 91 joints in 72 patients (70 joints in 55 females and 21 joints in 17 males). Overall mean treatment duration was 11.5 months (range, 9–14 months) for ARS. Eighteen patients underwent subsequent orthodontic treatment for irreversible occlusal changes to further achieve a stable occlusion and a new jaw position.

### Clinical symptoms

The VAS scores for pain and disability in daily life showed significant improvement following treatment. Mean VAS score for pain decreased from 3.89 at T0 to 2.23 at T2, and 1.37 at T3; compared with T0, this change was statistically significant (*P* < 0.001). There was also a significant difference for VAS quantitative disability score in daily life after functional treatment. While a total of 82 joints (90.11%) had TMJ clicking before treatment, only 9 (9.89%) had TMJ noises at T2, and 11 (12.09%) at T3; compared with T0, this decrease was statistically significant (*P* < 0.001). However, there was no significant difference in MIO, protrusive and lateral excursion following ARS treatment (Table 1).

**Table 1:** Comparison of clinical data of 72 patients (91 joints) at the various time points.

Evaluation time point	T0	T2	T3	*P*	Multiple Compaisons
TMJ pain (VAS)	3.89 ± 1.80	2.23 ± 1.77	1.37 ± 1.57	<0.001*	T1 > T2 > T3
Disability in daily life (VAS)	4.42 ± 1.53	3.66 ± 1.64	2.50 ± 1.38	<0.001*	T1 > T2 > T3
TMJ clicking	82/91	9/91^†^	11/91^†^		
No. joints (%)	(90.11%)	(9.89%)	(12.09%)		
MIO (mm)	40.61 ± 4.88	39.98 ± 4.98	39.45 ± 5.53	0.066	
PE (mm)	7.02 ± 1.37	7.27 ± 1.15	7.10 ± 1.52	0.429	
LLE (mm)	8.16 ± 1.68	8.59 ± 2.32	8.39 ± 1.54	0.191	
RLE (mm)	7.90 ± 1.30	8.26 ± 1.79	8.42 ± 1.94	0.073	

*Repeated measures analysis of variance test.

^†^McNemar Chi-square (significant at the level of *P* < 0.01, compared with T0).

### MRI findings

**Figure 6:**
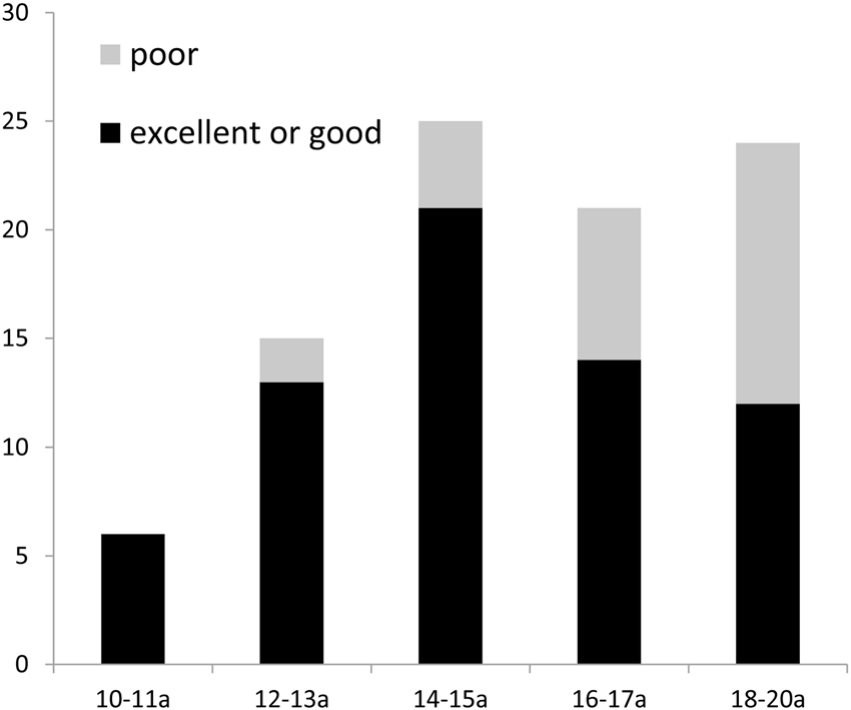
Joints with successful and unsuccessful ARS disc capture confirmed by MRI at T3 in different age groups. Unsuccessful joints: 2 cases in 12–13a group; 4 cases in 14–15a group; 7 cases in 16–17a group; 12 cases in 18–20a group.

MRI at T2 showed complete disc recapture with “double contour” images of the condyle in 64.83% (59 of 91 joints), indicating excellent outcomes. Twenty-five joints (27.47%) showed partially captured discs, indicating good outcome. The remaining 7 joints (7.69%) showed no evidence of disc capture at all and were judged as treatment failures. At follow-up at the end of 12 months (T3), MRI showed excellent outcome in 39 joints (42.86%), good outcome in 27 joints (29.67%), and treatment failure in 25 joints (27.47%). Thus, the total success rate decreased from 92.31% at T2 to 72.53% at T3 (Table 2). Age distribution of patients with successful and unsuccessful joints is shown in Fig. 6. The unsuccessful splint disc capture was mainly observed in late puberty, especially for patients over 16 years old.

**Table 2:** MRI assessment of ARS for treating DDwR (72 patients, 91 joints).

Evaluation period	Excellent (No. of joints)	Good (No. of joints)	Poor (No. of joints)	Treatment efficacy (%)
T2	59	25	7	92.31
T3	39	27	25	72.53

### Comparison of clinical evaluation versus MRI results

Table 3 shows the results of comparison of clinical evaluation with the results of MRI assessment. Clinically, splint capture was successful in 72 (79.12%) of the 91 joints. In the remaining 14 (15.38%) joints, the splint capture was considered unsuccessful by clinical criteria. MRI and clinical examination showed agreement in 75.82% of the joints. Clinical evaluation resulted in 14 false negatives (56.00%; 14 of 25) and 8 false positives (12.12%; 8 of 66). The positive predictive value was 57.90% and the negative predictive value was 80.56%.

**Table 3:** Comparison of the result of clinical evaluation versus MRI evaluation of splint capture at 12-month follow-up (72 patients, 91 joints).

Results of the clinical assessment	Results of MRI assessment	
	Excellent or good (No. of joints)	Poor (No. of joints)
Successful	0 58	0 14
Unsuccessful	0 8	0 11

For the clinical assessment with MRI, false negative, 14/25 = 56.00%; false positive, 8/66 = 12.12%; positive predictive value, 11/19 = 57.90%; negative predictive value, 58/72 = 80.56%; accuracy, 69/91 = 75.82%.

## Discussion

The findings of this study revealed that bite jumping with the ARS appliance resulted in a relatively stable repositioning of the disc in the majority of the subjects and improved TMJ symptoms 12 months after treatment (without ARS insertion).

TMJ disease is known to be much more common in women than in men; this seems true in our study sample also. The reasons for this difference in incidence of TMJ disease have not yet been elucidated, but biomechanical, physiological, genetic, and hormonal factors all possibly have a role^22^. One hypothesis is that the presence of oestrogen receptors in the TMJ of women alters metabolic functions and increases ligament laxity^23^.

In the present study, TMJ pain was significantly reduced after functional treatment; this was in agreement with Lundh *et al*.^24^, who credited it to the healing of discal elongation. We think the decrease in pain might also be related to the reduction in TMJ loading, which is associated with considerable increase in the posterosuperior space, improvement in occlusion, and a balanced distribution of muscle force^6^ Subjective assessment after treatment also showed significant improvement in jaw function. TMJ clicking, which was present in 90.11% of joints before treatment was seen in only 12.09% of joints at 12 months after treatment. This is concordant with the findings of Fayed *et al*.^25^ and Simmons and Gibbs^26^, who proposed that the elimination of clicking might be due to the establishment of a harmonious relationship between the condylar head, articular disc, and glenoid fossa.

In our research, MRI evaluation showed a success of 92.31% after ARS treatment, but this decreased to 72.53% at 12 months after treatment. In Moloney and Howard’s study^27^, they reported a 70% success rate after 1 year, a 53% success rate after 2 years, and only a 36% success rate after 3 years after treatment with ARS. Okeson^28^ reported that 75% of the patients had no joint pain and 66% had a return of joint sounds after 2.5 years.It should be note that only clinical outcomes were evaluated in these studies. Fayed *et al*.^25^ reported disc recapture (confirmed by MRI) in 25% of their DDwR patients who were treated with ARS. Chen *et al*.^17^ reported that only 40.6% (13/32) of the joints were maintained in the normal disc-condylar relationship 12 months after ARS treatment. The apparent discrepancy in these results may be attributed to differences in case selection and degree of TMJ arthropathy. Factors such as age, gender, and illness duration and treatment duration and criteria for success may be influence treatment results in patients with DDwR. In addition, we speculated that anteriorly displaced discs may not be really captured with the insertion of the appliance at initial splint therapy. Therefore, we excluded joints if unsuccessful disc capture occurred with bite registration prior to functional appliance therapy, which could provide more objective and accurate outcomes for effectiveness. We also found that patients in late puberty with unsuccessful splint disc capture, thus poor functional appliance treatment results or relapse seems relevant to the age of patients at initial visit. Strong correlation between age and functional treatment has been reported^29,30^. The factors which influenced successful or non-successful splint disc capture by the insertion of a disc repositioning appliance will be further discussed in future.

It is important to emphasize Class II malocclusion is corrected after insertion of ARS as a functional mandibular advancement device, while mandible protrusion could further improve the possibility of disc reduction, or the achievement of a physiology relationship between the disc and the condyle. In this study, MRI revealed double contours of the condyle in 39 joints 1 year after ARS treatment. Ruf and Pancherz^31,32^ have also documented condylar remodelling following herbst therapy. This may be attributable to the advancement of the mandible and the disc repositioning associated with the increased posterosuperior joint space, which minimizes joint loading^33^. Tensile stress on the condylar cartilage, in turn, would cause condylar remodelling. Thus, active condylar shape modification may be expected as an adaptive mechanism. Meanwhile, with mandibular adaptive growth, Class II malocclusion in the period of puberty can also be corrected after functional appliance treatment, which helps to stabilize the recaptured disc on the head of the condyle. Furthermore, a cephalometric investigation of changes in the dentofacial morphology and effective condylar growth will be performed to analyse the mechanisms contributing to the TMJ response upon splint treatment and a prospective clinical trial including patients without ARS treatment as a control group will also be added in our next research.

When compared with the results of MRI, clinical evaluation showed an accuracy rate of 75.82%, with the rate of the false positives was 12.12%. Our results also showed that 57.90% of the joints had confirmed disc displacement, and 80.56% of those were correctly identified. This indicated that when an unsuccessful clinical result was judged, it was a true failure about 57.90% of the time and if there was a success clinical result, 80.56% was real success.

## Conclusions

Seventy-two juvenile patients with 91 joints (DDwR) were treated with ARS therapy and a success rate was 92.31% at the end of treatment and 72.53% after 12 months. These outcomes indicate that the stability of normal disc-condylar relationship could be maintained in the majority of joints, especially for patients in early puberty. In this study, ARS used as a functional appliance could help re-establish a normal disc-condylar relationship and simultaneously correcting Class II skeletal malocclusions by enhancing condylar adaptive remodelling and mandibular growth. However, a larger sample with longer follow-up are also required to fully determine the long-term efficacy of ARS.
